# Naoxintong capsule as adjunctive therapy in ACS: a hypothesis-generating open-label randomized trial

**DOI:** 10.3389/fphar.2026.1848036

**Published:** 2026-08-04

**Authors:** Liyuan Yu, Weihang Peng, Lulu Wu, Hairong Cai, Jing Zeng, Bojun Chen

**Affiliations:** 1 The Second Clinical Medical School of Guangzhou University of Chinese Medicine, Guangzhou, China; 2 Guangdong Provincial Hospital of Traditional Chinese Medicine, Guangzhou, China; 3 Affiliated TCM Hospital of Guangzhou Medical University, Guangzhou, China; 4 School of Traditional Chinese Medicine, Guangdong Pharmaceutical University, Guangdong, China; 5 State Key Laboratory of Traditional Chinese Medicine Syndrome, Guangdong Provincial Key Laboratory of Research on Emergency in Traditional Chinese Medicine, Clinical Research Team of Prevention and Treatment of Cardiac Emergencies with Traditional Chinese Medicine, Guangzhou, China

**Keywords:** acute coronary syndrome, anxiety, depression, ethnopharmacology, major adverse cardiovascular events, naoxintong capsule, traditional Chinese medicine

## Abstract

**Objective:**

To evaluate, in an exploratory setting, the safety of adjunctive Naoxintong Capsule (NXT) added to standard secondary prevention in patients with acute coronary syndrome (ACS), and to describe between-group differences in major adverse cardiovascular events (MACE), psychological state, angina-related quality of life, lipid profile, cardiac function, and traditional Chinese medicine (TCM) syndrome measures.

**Materials and methods:**

In this open-label, multicenter, exploratory randomized controlled trial, 170 ACS patients were randomized 1:1 to standard secondary prevention alone (control, n = 85) or combined with NXT (n = 85) for 12 months. The primary outcome was 12-month MACE (cardiac death, nonfatal myocardial infarction, urgent revascularization, ischemic stroke), ascertained by treating clinicians without an independent blinded events committee. Secondary outcomes included SAS, SDS, SAQ, lipid profile, echocardiographic parameters, TCM syndrome score, and safety indices. The primary analysis was complete-case; multiplicity was not pre-specified, and all secondary P values are nominal.

**Results:**

Of 170 randomized participants, 152 completed 12-month follow-up (NXT n = 78; control n = 74; attrition 10.6%, balanced between arms). MACE was recorded in 8/78 (10.3%) in the NXT arm vs. 13/74 (17.6%) in control (relative risk 0.58; 95% CI 0.26–1.33; P = 0.19; not statistically significant, 95% CI included the null). A worst-case sensitivity check assigning all NXT-arm losses as events and all control-arm losses as non-events yielded 17.6% vs. 15.3%, indicating that the direction of the point estimate was not robust to missing-outcome assumptions. Nominally larger reductions in the NXT arm were observed for SAS (−8.75 vs. −3.75) and SDS (−10.00 vs. −3.75); nominally larger changes were also observed across SAQ domains, lipid parameters (TC, TG, LDL-C), and echocardiographic indices (LVEF, LVEDD, LVESD), all reported as nominal descriptive findings. Safety laboratory parameters did not differ between groups, and no serious adverse events occurred.

**Conclusion:**

In this open-label exploratory trial, adjunctive NXT was not associated with a statistically significant reduction in MACE, and the direction of the estimate was not robust to missing-data assumptions. Between-group differences in secondary outcomes are hypothesis-generating only, given the open-label design, unblinded event ascertainment, absence of multiplicity adjustment, and complete-case analysis of a rare-event endpoint. Adequately powered, placebo-controlled, double-blind trials with independent blinded event adjudication are needed.

**Clinical Trial Registration:**

Registered on July 22, 2020, with the registration number ChiCTR2000034871 at the Chinese Clinical Trial Registry.

## 1 Introduction

Acute coronary syndrome (ACS) remains a leading global cause of cardiovascular-related disability and mortality (Roman et al., 2025). Although the application of percutaneous coronary intervention and standardized secondary prevention strategies, such as antiplatelet agents and statins, has significantly improved the survival rate of ACS patients, survivors still face a high residual risk of major adverse cardiovascular events (MACE) (Spirito et al., 2024; Tannu et al., 2025; Lee et al., 2025). Furthermore, the high prevalence of psychological disorders like anxiety and depression, as well as refractory angina, significantly impairs patients’ quality of life and long-term prognosis, presenting a core challenge in contemporary clinical management (Carney et al., 2024). While modern clinical guidelines emphasize comprehensive management, existing treatment regimens still have limitations in simultaneously improving patients’ psychological status and physiological parameters, such as cardiac function and lipid metabolism (Claessen et al., 2020).

Traditional Chinese Medicine (TCM), characterized by its multi-target and holistic regulatory approach, offers a potential strategy for the comprehensive management of ACS (Pan et al., 2025; Li et al., 2025; Liu et al., 2024). Naoxintong Capsule (NXT), a compound Chinese herbal formulation, is known for its effects in “promoting blood circulation, removing stasis, tonifying Qi, and unblocking collaterals.” (Zhang et al., 2024; Yu et al., 2024; Wan et al., 2024). Preclinical research suggests its potential in improving myocardial perfusion and anti-atherosclerosis (Xiao et al., 2024; Hu et al., 2021; Wang et al., 2018). However, there is a lack of high-level evidence from rigorous randomized controlled trials (RCTs) regarding whether NXT, as an adjunct to conventional therapy, can provide synergistic benefits by further improving hard endpoints (e.g., MACE) and multi-dimensional psychological-physiological indicators in ACS patients.

Therefore, this study aimed to systematically evaluate the efficacy and safety of Naoxintong Capsule added to standard secondary prevention in improving multiple dimensions—including the incidence of MACE, psychological status, angina symptoms, cardiac function, and lipid metabolism—in ACS patients through a randomized controlled trial. The findings are intended to provide a scientific basis for optimizing integrated management strategies for ACS.

## 2 Materials and methods

### 2.1 Study design and oversight

This multicenter, randomized, controlled, open-label, parallel-group trial was conducted at three centers: Guangdong Provincial Hospital of Traditional Chinese Medicine (coordinating center), Chenzhou First People’s Hospital, and Foshan Sanshui District People’s Hospital. The protocol was approved by the institutional ethics committee in 2020 and prospectively registered with the Chinese Clinical Trial Registry on 22 July 2020 (ChiCTR2000034871). Because of the COVID-19 pandemic and project preparation, recruitment and the 12-month intervention and follow-up took place between June 2022 and February 2024. As randomization used a single computer-generated sequence without stratification by center, participants were pooled across sites for both randomization and analysis; the coordinating center enrolled the majority of participants, with the remainder recruited from the two collaborating sites. The trial adhered to the Declaration of Helsinki and Good Clinical Practice, and written informed consent was obtained from all participants. Reporting follows the CONSORT 2010 statement.

### 2.2 Participants

#### 2.2.1 Diagnostic criteria for ACS

ACS included STEMI, NSTEMI, and UA. STEMI was diagnosed according to the Fourth Universal Definition of Myocardial Infarction (2018) as a rise and/or fall in cardiac troponin (cTn), with at least one value above the 99th percentile upper reference limit, together with evidence of myocardial ischemia, including ischemic symptoms, new ST-segment elevation or pathological Q waves, imaging evidence of ischemia, or angiographic evidence of coronary thrombus. NSTEMI and UA were diagnosed according to the 2020 guideline for non-ST-segment elevation ACS. NSTEMI was defined by ischemic symptoms without persistent ST-segment elevation, accompanied by ischemic electrocardiographic changes and elevated cTn. UA was defined by ischemic symptoms without persistent ST-segment elevation and without myocardial necrosis, with cTn not exceeding the 99th percentile upper reference limit (Thygesen et al., 2018; Collet et al., 2021).

#### 2.2.2 Inclusion criteria

Adults (18–75 years) with ACS (STEMI, NSTEMI, or unstable angina) per contemporary definitions; NYHA I–II; TCM diagnosis of Qi deficiency and blood stasis syndrome according to recognized TCM diagnostic criteria for coronary heart disease/acute coronary syndrome, independently assessed by two senior TCM practitioners, with disagreements resolved by consensus; able to complete SAS/SDS; provided informed consent.

#### 2.2.3 Exclusion criteria

NYHA III–IV or cardiogenic shock; severe hepatic/renal dysfunction or hematologic/autoimmune disorders; active malignancy or life expectancy <1 year; pregnancy/lactation; participation in another interventional study within 3 months; hypersensitivity to NXT components.

#### 2.2.4 Withdrawal criteria

Discontinuation of study medication, intercurrent events precluding follow-up, or loss to follow-up (reasons recorded).

### 2.3 Randomization, allocation concealment, and blinding

Randomization and blinding: Participants were assigned in a 1:1 ratio according to a computer-generated randomization sequence without stratification by center. Allocation concealment was ensured using sealed, opaque, sequentially numbered envelopes prepared in advance and opened sequentially at assignment. Because a matched placebo was not feasible, the trial was open-label. Participants, treating clinicians, and outcome assessors were aware of treatment assignment. Statisticians performing the pre-specified analyses were blinded to group labels until the analysis dataset was locked. The implications of unblinded outcome assessment - particularly for subjective and investigator-measured outcomes - are addressed in the Limitations.

### 2.4 Interventions

#### 2.4.1 Control group

Participants received standard secondary prevention therapy for coronary heart disease, including aspirin (100 mg daily), a P2Y_12_ inhibitor (clopidogrel 75 mg daily or ticagrelor 90 mg twice daily), β-blockers (e.g., metoprolol succinate), and statins (atorvastatin calcium 20–40 mg daily). Concomitant treatments for hypertension, diabetes, or arrhythmia were administered per guideline recommendations.

#### 2.4.2 NXT group

In addition to the standard regimen, participants received Naoxintong Capsule (Shaanxi Buchang Pharmaceutical Co., Ltd.; 0.4 g per capsule), four capsules per dose, three times daily after meals for 12 months.

Use of other TCM formulations with similar pharmacological actions (e.g., Qi-tonifying or blood-activating decoctions) was not permitted during the trial.Adherence to the study intervention was monitored during scheduled follow-up visits by routine patient inquiry and return of empty medication boxes. These procedures were used for treatment management during the trial, although a formal quantitative adherence dataset was not prospectively established. Because quantitative adherence data (pill counts, dosing diaries) and detailed concomitant-medication titration data (including statin dose adjustment over 12 months) were not systematically collected, the influence of differential adherence to background therapy between arms cannot be quantified.

### 2.5 Herbal material authentication and quality control (per ethnopharmacology best practices)

Naoxintong Capsule is a marketed proprietary Chinese patent medicine approved by the NMPA (Approval No. Z20025001; Shaanxi Buchang Pharmaceutical Co., Ltd.), manufactured under GMP and monographed in the Chinese Pharmacopoeia (2020 Edition, Vol. I). The specification is 0.4 g per capsule, containing a mixture of dry extract and fine herbal powder as defined by the monograph. Raw-material authentication, batch-release testing, and retention sampling were performed by the manufacturer per Pharmacopoeia standards (macroscopic, microscopic, and TLC/HPLC identification, plus marker-constituent assay). The product was administered in its original commercial packaging without modification.

#### 2.5.1 Composition (Chinese name → Latin binomial [plant part])

Huangqi → *Astragalus membranaceus* (Fisch.) Bunge [Radix]; Chishao → *Paeonia* spp. (Radix rubra); Danshen → *Salvia miltiorrhiza* Bunge [Radix/Rhizoma]; Danggui → *Angelica sinensis* (Oliv.) Diels [Radix]; Chuanxiong → *Ligusticum chuanxiong* Hort [Rhizoma]; Taoren → *Prunus persica* (L.). Batsch [Semen]; Honghua → *Carthamus tinctorius* L. [Flos]; vinegar-processed Ruxiang → *Boswellia* spp. [Resina]; vinegar-processed Moyao → *Commiphora* spp. [Resina]; Jixueteng → *Spatholobus suberectus* Dunn [Caulis]; Niuxi → *Achyranthes bidentata* Blume [Radix]; Guizhi → *Cinnamomum cassia* (L.) J. Presl [Ramulus]; Sangzhi → *Morus alba* L. [Ramulus]; Dilong → *Pheretima aspergillum* (E. Perrier); Quanxie → *Buthus martensii* Karsch; Shuizhi → *Hirudo* spp. [Animalia].

As the intervention was a licensed marketed product rather than investigator-prepared material, independent voucher specimens were not deposited.

### 2.6 Outcomes and measurements

Participants were followed at baseline and at 3, 6, and 12 months. Interim follow-up visits were used for routine clinical assessment and safety monitoring, while the 12-month data were used for the primary efficacy analysis.

#### 2.6.1 Primary outcome

12-month MACE was defined as a composite of cardiac death, nonfatal myocardial infarction, urgent revascularization, and ischemic stroke. Cardiac death was defined as death resulting from acute myocardial infarction, heart failure, arrhythmia, sudden unexplained death, or other cardiovascular causes. Nonfatal myocardial infarction was defined according to the Fourth Universal Definition of Myocardial Infarction, requiring a rise and/or fall in cardiac troponin with at least one value above the 99th percentile upper reference limit, together with evidence of myocardial ischemia. Urgent revascularization was defined as unplanned PCI or CABG prompted by recurrent ischemic symptoms or objective evidence of myocardial ischemia. Ischemic stroke was defined as a new focal neurological deficit of presumed vascular origin lasting more than 24 h, with imaging confirmation when available.

Event ascertainment and adjudication. MACE components were ascertained by the treating clinicians at each participating site on the basis of routine clinical assessment during scheduled follow-up visits, spontaneous patient contact, and review of medical records. An independent blinded clinical events committee was not established for this trial. Because the treating clinicians were aware of allocation, the ascertainment of subjective components - particularly urgent revascularization - was not blinded; the potential for detection bias is addressed in the Discussion.

#### 2.6.2 Secondary outcomes

##### 2.6.2.1 Psychological state

Anxiety and depression were evaluated using the Self-Rating Anxiety Scale (SAS) and Self-Rating Depression Scale (SDS). Each scale consists of 20 items rated on a four-point Likert scale. Standardized scores were derived by multiplying raw scores by 1.25, with higher scores indicating greater symptom severity.

##### 2.6.2.2 Angina-related quality of life

Measured using the Seattle Angina Questionnaire (SAQ), which includes five domains: Physical Limitation (PL), Angina Stability (AS), Angina Frequency (AF), Treatment Satisfaction (TS), and Disease Perception (DS). Each domain is scored from 0 to 100, where higher values indicate better quality of life.

##### 2.6.2.3 TCM syndrome score and efficacy

The TCM syndrome score was determined using a validated 0–3 Likert scale for key clinical manifestations; assessors were not blinded to allocation. Therapeutic efficacy was classified as markedly effective (≥70% reduction in total score), effective (≥30%), or ineffective (<30%).

##### 2.6.2.4 Laboratory indices

Fasting lipid profile (TC, TG, HDL-C, LDL-C) and echocardiographic parameters (LVEF, LVEDD, LVESD, FS) were measured before and after treatment. Echocardiographic images were acquired and interpreted at each participating site without independent blinded core-laboratory review.

##### 2.6.2.5 Safety outcomes

Routine hematology, liver (ALT, AST), and renal function (serum creatinine) tests were performed at baseline and after 12 months of treatment. All adverse events were recorded, and causality was independently evaluated by investigators.

Copies of all psychometric assessment instruments, including the Self-Rating Anxiety Scale (SAS), Self-Rating Depression Scale (SDS), Seattle Angina Questionnaire (SAQ), and the TCM syndrome score form, are provided in Supplementary Table S1 for reference and reproducibility.

### 2.7 Sample size estimation

The sample size was estimated using PASS version 21.0 (NCSS, LLC, United States), based on preliminary data indicating a 32% MACE incidence in the control group versus 11% in the Naoxintong Capsule (NXT) group. Assuming a two-sided α of 0.05 and a power (1–β) of 0.90, the minimum required sample size was 76 participants per group.Considering a 10% dropout rate, the total enrollment target was 170 participants (85 per group) to ensure adequate statistical power.

A detailed report of the PASS sample size calculation is provided in Supplementary Methods S1 to enhance methodological transparency and reproducibility. The observed control-arm MACE rate was substantially lower than this *a priori* assumption; the implications for interpretation of the primary endpoint are addressed in the Limitations.

### 2.8 Statistical analysis

Statistical analyses were conducted using SPSS version 27.0.Normality was assessed using the Shapiro–Wilk test. Data conforming to normal distribution were expressed as mean ± standard deviation (SD) and analyzed using the independent-samples t-test (between groups) or paired t-test (within groups).Non-normally distributed data were expressed as median (interquartile range, IQR) and analyzed using the Mann–Whitney U test or Wilcoxon signed-rank test. Categorical variables were compared using the chi-square test or Fisher’s exact test where appropriate.For key outcomes, relative risk (RR) and mean difference (MD) with 95% confidence intervals (CI) were calculated. All tests were two-tailed, with P < 0.05 indicating statistical significance for the primary endpoint.

Handling of multiple secondary outcomes. The primary endpoint was the 12-month MACE composite. Secondary outcomes spanned psychological, symptom, laboratory, echocardiographic, and TCM-syndrome domains. No adjustment for multiplicity was pre-specified; accordingly, all secondary P values are reported as nominal and are interpreted as descriptive and hypothesis-generating rather than confirmatory.

Handling of missing outcome data. The primary analysis was a complete-case analysis restricted to participants who completed 12-month follow-up. Given the small number of observed events relative to losses to follow-up, imputation-based analyses would be dominated by modeling assumptions rather than by observed data; formal multiple-imputation and tipping-point analyses were therefore not performed. A worst-case sensitivity check for the MACE endpoint is reported in Results 3.2.

## 3 Results

### 3.1 Participant flow and baseline characteristics

**FIGURE 1:**
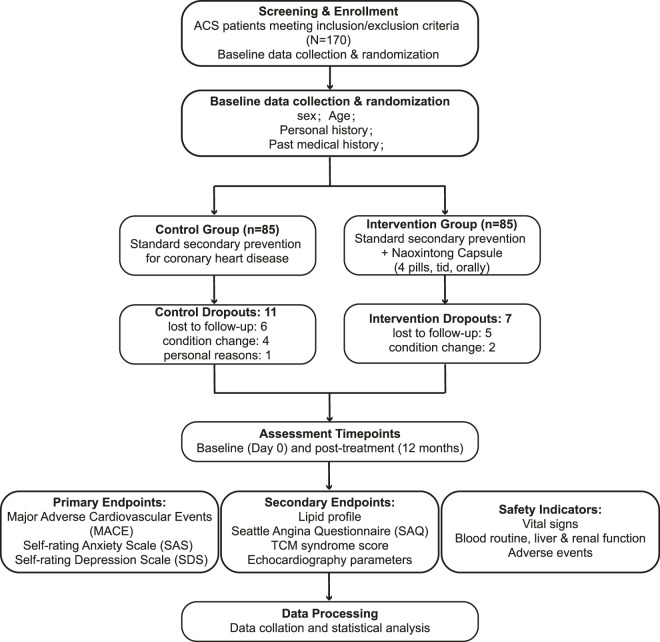
Participant flow (CONSORT-style).

From June 2022 to February 2024, 170 patients were randomized; 152 completed follow-up (Naoxintong n = 78; control n = 74). Attrition was balanced and unrelated to the intervention (total 10.6%): Naoxintong arm 7 (lost to follow-up 5; clinical condition change 2); control arm 11 (lost to follow-up 6; condition change 4; personal reason 1). See CONSORT diagram (Figure 1). Baseline demographics, risk factors, revascularization rates (PCI), psychometrics, lipids, and echocardiography were comparable (all *P* > 0.05; Table 1).

**TABLE 1:** Baseline characteristics of participants.

Characteristic	NXT group (n = 78)	Control group (n = 74)	$t/Z/\chi^{2}$	P
Sex, no. (%)	​	​	-	-
Men	62 (79.49)	59 (79.73)	0.001	0.970
Women	16 (20.51)	15 (20.27)		
Age, (*M*(*P*_25_, *P*_75_)), y	60 (51, 70)	61.5 (52, 68.25)	−0.131	0.896
Medical history, no. (%)				
Family history of hypertension	43 (55.13)	41 (55.41)	0.001	0.973
Family history of coronary artery disease	31 (39.74)	30 (40.54)	0.008	0.930
Family history of diabetes mellitus	39 (50.00)	32 (43.24)	0.696	0.404
Smoking history	44 (56.41)	41 (55.41)	0.016	0.901
Drinking history	40 (51.28)	42 (56.76)	0.458	0.499
Body mass index, mean (SD)^a^	23.98 (2.88)	23.34 (2.03)	−1.585	0.115
Percutaneous coronary intervention (n, %)	59 (75.64)	50 (67.57)	1.220	0.269
Self-rating anxiety scale, median (IQR), score^b^	50 (43.44, 55)	48.75 (43.75, 56.56)	−0.133	0.894
Self-rating depression scale, median (IQR), score^b^	46.25 (38.44, 57.81)	44.38 (35.94, 57.81)	−0.681	0.496
Seattle Angina questionnaire, median (IQR), score^c^	​	​	-	-
Physical limitation	17.78 (8.34, 51.67)	15.56 (8.34, 47.23)	−0.541	0.589
Angina stability	25 (0, 25)	25 (0, 25)	−0.325	0.745
Angina frequency	40 (10, 70)	40 (10, 70)	−0.045	0.964
Treatment satisfaction	52.94 (29.41, 77.94)	52.94 (29.41, 76.47)	−0.12	0.904
Disease perception	33.33 (16.67, 33.33)	33.33 (16.67, 41.67)	−0.349	0.727
TCM symptom quantification score, median (IQR), score	15 (14, 16)	16 (14, 16.25)	−1.658	0.097
Total cholesterol level, median (IQR), mmol/L	4.92 (4.31, 5.53)	4.78 (3.97, 5.51)	−0.909	0.364
Triglyceride level, median (IQR), mmol/L	1.57 (1.18, 2.13)	1.6 (1.17, 1.90)	−0.636	0.525
HDL cholesterol level, median (IQR), mmol/L	0.98 (0.84, 1.09)	0.91 (0.79, 1.09)	−0.948	0.343
LDL cholesterol level, median (IQR), mmol/L	3.35 (2.66, 3.75)	2.99 (2.37, 3.67)	−1.438	0.151

a Calculated as weight in kilograms divided by height in meters squared.

b SAS, Self-Rating Anxiety Scale; SDS, Self-Rating Depression Scale. Scores for both scales were presented as standard scores (SAS, range: 25–100; SDS, range: 25–100).

c SAQ, Seattle Angina Questionnaire. Scores range from 0 to 100, with higher scores indicating better health status and fewer limitations.

### 3.2 Primary outcome: major adverse cardiovascular events (MACE)

**FIGURE 2:**
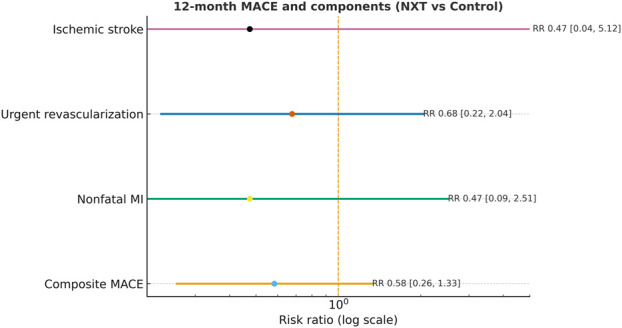
Relative risks (RRs) for MACE and component endpoints at 12 months.

At 12 months, MACE occurred in 8/78 (10.3%) in NXT vs. 13/74 (17.6%) in control (RR 0.58; *95% CI* 0.26–1.33; *P* = 0.19; the between-group difference did not reach statistical significance). Individual components numerically favored NXT: nonfatal MI 2.6% vs. 5.4% (RR 0.47; *95% CI* 0.09–2.51), urgent revascularization 6.4% vs. 9.5% (RR 0.68; *95% CI* 0.22–2.04), ischemic stroke 1.3% vs. 2.7% (RR 0.47; *95% CI* 0.04–5.12). No cardiac deaths were recorded in either arm. All confidence intervals for the composite endpoint and its components crossed unity. The numerical between-group difference in the composite was driven predominantly by urgent revascularization - the component most susceptible to ascertainment bias in an open-label trial without an independent blinded clinical events committee - and this pattern is consistent with, and cannot be distinguished from, detection bias. Accordingly, no inference regarding a treatment effect of NXT on MACE or any of its components is drawn. Event counts and relative risks are presented in Figure 2 and Table 2.

**TABLE 2:** Primary outcome (MACE) and component events at 12 months [n (%)].

Event	NXT group (n = 78)	Control group (n = 74)	RR (95% CI)	χ^2^	P
Non-fatal myocardial infarction	2 (2.56)	4 (5.40)	0.47 (0.09–2.51)	0.809	0.433a
Urgent revascularization (PCI or CABG)	5 (6.41)	7 (9.50)	0.68 (0.22–2.04)	0.486	0.486
Acute ischemic stroke	1 (1.28)	2 (2.70)	0.47 (0.04–5.12)	0.396	0.613a
Total MACE events	8 (10.25)	13 (17.60)	0.58 (0.26–1.33)	1.705	0.192

RR, relative risk; CI, confidence interval; P values from χ^2^ test unless otherwise indicated; a Fisher’s exact test (two-sided).

Given (i) the substantially lower-than-anticipated control-arm event rate (17.6% vs. the 32% assumed in the sample-size calculation), (ii) attrition of 10.6% relative to only 21 total events, and (iii) the open-label design without blinded event adjudication, the MACE analysis should be regarded as inconclusive. A worst-case sensitivity check assuming all NXT-arm losses experienced MACE and all control-arm losses did not, would yield rates of 15/85 (17.6%) vs. 13/85 (15.3%), illustrating that the direction of the point estimate is not robust to missing-outcome assumptions.

### 3.3 Secondary outcomes

Because multiple secondary outcomes were assessed across psychological, symptom, laboratory, echocardiographic, and TCM-syndrome domains, and because no adjustment for multiplicity was pre-specified, all P values reported below are nominal and descriptive. Given the open-label design and absence of placebo control, the differences described in this section cannot be attributed to a pharmacological effect of NXT and should be considered hypothesis-generating only, intended to inform the design of future adequately powered, blinded trials.

#### 3.3.1 Psychological and quality-of-life outcomes

##### 3.3.1.1 Psychological status

Nominally larger reductions were observed in the NXT arm for ΔSAS (median difference 5.00 points; *95% CI* 3.75–7.50; *P* < 0.001) and ΔSDS (6.25 points; *95% CI* 3.75–7.50; *P* < 0.001). Reductions from baseline were observed in both arms (nominal *P* < 0.001, Table 3).

**TABLE 3:** Comparison of Self-Rating Anxiety Scale (SAS) and Self-Rating Depression Scale (SDS) scores before and after treatment [median (P25, P75)].

Outcome	Time point	NXT group (n = 78)	Control group (n = 74)	*Z*	*P*
SAS	Baseline	50 (43.44, 55.00)	48.75 (43.75, 56.56)	−0.133	0.894
	Post-treatment	40 (33.75, 45.63)**	45 (38.75, 54.06)**	−3.497	<0.001
	Δ (baseline − post)	8.75 (5, 12.5)	3.75 (2.50, 5)	−7.062	<0.001
SDS	Baseline	46.25 (38.44, 57.81)	44.38 (35.94, 57.81)	−0.681	0.496
	Post-treatment	36.25 (31.25, 45.00)**	39.38 (33.44, 54.06)**	−2.020	0.043
	Δ (baseline − post)	10 (5, 13.75)	3.75 (2.50, 5)	−6.621	<0.001

Data are presented as median (P25, P75). **P < 0.001 vs. baseline within the same group (Wilcoxon signed-rank test).**

SAS, between-group difference on ΔSAS (trial − control): Hodges–Lehmann median difference = 5.00 points (95% CI, 3.75–7.50); Mann–Whitney U, Z = 7.062, P < 0.001.

SDS, between-group difference on ΔSDS (trial − control): Hodges–Lehmann median difference = 6.25 points (95% CI, 3.75–7.50); Mann–Whitney U, Z = 6.621, P < 0.001.

Δ denotes baseline − post-treatment (higher values indicate greater improvement).

Abbreviations: SAS, Self-Rating Anxiety Scale; SDS, Self-Rating Depression Scale.

##### 3.3.1.2 Angina-related quality of life (SAQ)

Nominally greater improvements were observed in the NXT arm across domains—PL, AS (P = 0.028), AF, TS, and DS—with Hodges–Lehmann median differences of 11.11 (PL), 0.00 (AS; 95% CI 0.00–25.00), 20.00 (AF), 5.88 (TS), and 8.34 (DS); all nominal *P* ≤ 0.008 except AS as noted (Table 4).

**TABLE 4:** Comparison of Seattle Angina Questionnaire (SAQ) domain scores before and after treatment [median (P25, P75)].

Outcome	Time point	NXT group (n = 78)	Control group (n = 74)	*Z*	*P*
PL	Baseline	17.78 (8.34, 51.67)	15.56 (8.34, 47.23)	−0.541	0.589
	Post-treatment	58.89 (26.67, 72.78)**	46.67 (15.56, 57.78)**	−3.89	<0.001
	Δ (baseline − post)	22.22 (15.56, 31.11)	8.89 (4.45, 17.78)	−5.222	<0.001
AS	Baseline	25 (0, 25)	25 (0, 25)	−0.325	0.745
	Post-treatment	100 (75, 100)**	75 (75, 100)**	−3.75	<0.001
	Δ (baseline − post)	75 (50, 75)	50 (50, 75)	−2.202	0.028
AF	Baseline	40 (10, 70)	40 (10, 70)	−0.045	0.964
	Post-treatment	70 (60, 90)**	50 (20, 80)**	−3.826	<0.001
	Δ (baseline − post)	30 (20, 40)	10 (0, 20)	−6.106	<0.001
TS	Baseline	52.94 (29.41, 77.94)	52.94 (29.41, 76.47)	−0.12	0.904
	Post-treatment	82.35 (58.82, 94.12)**	64.71 (41.18, 94.12)**	−1.598	0.11
	Δ (baseline − post)	23.53 (5.88, 30.88)	17.64 (0, 23.53)	−2.641	0.008
DS	Baseline	33.33 (16.67, 33.33)	33.33 (16.67, 41.67)	−0.349	0.727
	Post-treatment	41.67 (33.33, 58.33)**	33.33 (25, 41.67)**	−4.623	<0.001
	Δ (baseline − post)	16.66 (8.34, 16.67)	0 (0, 8.34)	−6.951	<0.001

Data are presented as median (P25, P75). **P < 0.001 vs. baseline within the same group (Wilcoxon signed-rank test).**

Between-group difference on Δ scores (trial − control; Hodges–Lehmann median differences with 95% CI):

SAQ-PL: 11.110 (95% CI, 8.880–13.350); Mann–Whitney U, Z = 5.222, P < 0.001.

SAQ-AS: 0.000 (95% CI, 0.000–25.000); Mann–Whitney U, Z = 2.202, P 0.028.

SAQ-AF: 20.000 (95% CI, 10.000–30.000); Mann–Whitney U, Z = 6.106, P < 0.001.

SAQ-TS: 5.880 (95% CI, 0.000–11.760); Mann–Whitney U, Z = 2.641, P 0.008.

SAQ-DS: 8.340 (95% CI, 8.330–16.660); Mann–Whitney U, Z = 6.951, P < 0.001.

For SAQ, domains, Δ is defined as post − baseline (higher values indicate greater improvement). Abbreviations: SAQ, seattle angina questionnaire; PL, physical limitation; AS, angina stability; AF, angina frequency; TS, treatment satisfaction; DS, disease perception.

#### 3.3.2 Traditional Chinese Medicine (TCM) syndrome evaluation

##### 3.3.2.1 Quantitative scores

Compared with control, the trial group achieved a nominally greater reduction in the TCM syndrome quantitative score. The median change [Δ = baseline − post-treatment; median (P25, P75)] was 8 (7, 10) in the trial group versus 7 (5, 9) in controls (*Z* = 2.794, nominal *P* = 0.005). The between-group difference (trial − control; Hodges–Lehmann median difference) was 1.00 point (*95% CI* 0.00–2.00) (Table 5).

**TABLE 5:** Traditional Chinese Medicine (TCM) syndrome quantitative score before and after treatment [median (P25, P75)].

Outcome	Time point	NXT group (n = 78)	Control group (n = 74)	*Z*	*P*
TCM syndrome score	Baseline	15 (14, 16)	16 (14, 16.25)	−1.658	0.097
	Post-treatment	7 (6, 8)**	8 (7, 10)**	−4.498	<0.001
	Δ (baseline − post)	8 (7, 10)	7 (5, 9)	−2.794	0.005

Data are presented as median (P25, P75). **P < 0.001 vs. baseline within the same group (Wilcoxon signed-rank test).**

Between-group difference on Δ score (trial − control): Hodges–Lehmann median difference = 1.00 points (95% CI, 0.00–2.00); Mann–Whitney U, Z = 2.794, P = 0.005.

Δ is defined as baseline − post-treatment (higher Δ indicates greater improvement).

##### 3.3.2.2 Syndrome efficacy

**FIGURE 3:**
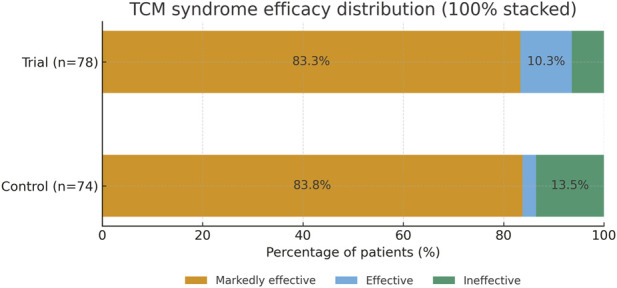
Distribution of TCM syndrome efficacy by group (100% stacked). Bars show within-group percentages rated markedly effective, effective, or ineffective in the trial (n = 78) and control (n = 74) groups. Overall comparison: Wilcoxon rank–sum/Mann–Whitney U test, Z = 2.219, P = 0.026.

The distribution of TCM-syndrome efficacy categories nominally differed between groups (Wilcoxon rank–sum/Mann–Whitney U test, *Z* = 2.219, nominal *P* = 0.026; see Figure 3). The trial group showed a nominal higher overall responder rate (markedly effective + effective: 73/78, 93.6%) than the control group (64/74, 86.5%) and a nominal lower proportion judged ineffective (5/78, 6.4% vs. 10/74, 13.5%). Category breakdown (trial vs. control) was: markedly effective 83.3% vs. 83.8%, effective 10.3% vs. 2.7%, ineffective 6.4% vs. 13.5%.

#### 3.3.3 Laboratory indicators

##### 3.3.3.1 Blood lipid profile

Compared with control, the trial group achieved nominally greater improvements in the lipid profile (see Supplementary Table S1). For TC, the reduction was larger in the trial group (Δ = baseline − post: median 1.45 vs. 0.61 mmol/L; *Mann–Whitney Z* = 3.934, nominal *P* < 0.001), with a between-group difference of +0.740 mmol/L (trial − control; Hodges–Lehmann median difference; *95% CI* 0.370–1.100). TG also decreased more in the trial group (0.39 vs. 0.18 mmol/L; *Z* = 2.061, nominal *P* = 0.039), corresponding to an effect of +0.260 mmol/L (*95% CI* 0.020–0.520). LDL-C showed a greater decline in the trial group (0.94 vs. 0.43 mmol/L; *Z* = 2.711, nominal *P* = 0.007), with an effect of +0.470 mmol/L (*95% CI* 0.130–0.820). In contrast, HDL-C increases were similar between groups (Δ = post − baseline: 0.23 ± 0.32 vs. 0.18 ± 0.33 mmol/L; mean difference [trial − control] +0.053 mmol/L, *95% CI* −0.050 to 0.157, t (150) = −1.013, nominal *P* = 0.313). Within-group changes from baseline were significant for most markers.

##### 3.3.3.2 Echocardiography

Compared with control, the trial group showed greater improvements in cardiac structure and function (see Supplementary Table S2). The increase in LVEF was larger in the trial group (Δ = post − baseline: median 8% vs. 4%; *Mann–Whitney Z* = 2.923, nominal *P* = 0.003), corresponding to a between-group difference of +4.0% (trial − control; Hodges–Lehmann median difference, *95% CI* +1.0 to +7.0). The reduction in LVEDD was also greater (7 vs. 4 mm; *Z* = 3.312, nominal *P* < 0.001), with an effect of +3.0 mm (*95% CI* +1.0 to +5.0). For LVESD, the decrease favored the trial group (8.13 ± 8.07 vs. 3.64 ± 6.59 mm; mean difference [trial − control] +4.49 mm, *95% CI* +2.14 to +6.85, t (Welch) < 0.001). Changes in FS were not significantly different between groups (5.53% ± 14.71% vs. 1.85% ± 12.28%, mean difference +3.68%, *95% CI* −0.65 to +8.02, nominal P = 0.095). Baseline values were comparable across groups.

##### 3.3.3.3 Safety outcomes

No between-group differences were observed in safety laboratory indices (see Supplementary Table S3). For the change from baseline (Δ = post - baseline), the Hodges–Lehmann median differences (trial - control) and *95% CIs* were: WBC -0.44 (−1.04 to 0.32), RBC +0.01 (−0.12–0.17), Hb +0.03 (−4.00–5.00), PLT −15.00 (−33.00 to 6.00), ALT −0.16 (−5.00 to 2.86), AST +1.00 (−4.00–4.15), and Cr +2.00 μmol/L (−1.00–9.99); corresponding two-sided P values were 0.222, 0.848, 0.891, 0.173, 0.723, 0.719, 0.248, respectively (all *P* > 0.17). Baseline values and post-treatment values were comparable between groups (all *P* ≥ 0.05). Collectively, the intervention did not adversely affect hematologic or hepatic/renal function.Furthermore, no serious or non-serious adverse events were reported in either group during the 12-month follow-up.

## 4 Discussion

This exploratory, open-label multicenter randomized trial evaluated adjunctive Naoxintong Capsule (NXT) in patients with ACS receiving standard secondary prevention. The trial did not demonstrate an effect of NXT on the primary composite endpoint of MACE; the observed between-group difference was not statistically significant, and - given the lower-than-anticipated control-arm event rate, attrition relative to the small number of events, absence of blinded event adjudication, and the open-label design - the MACE result is best interpreted as inconclusive rather than suggestive of benefit. Nominally larger changes in the NXT arm were observed for several patient-reported and intermediate outcomes, including psychological status (SAS/SDS), angina-related quality of life (SAQ), lipid parameters, and echocardiographic indices. Because these secondary analyses were not adjusted for multiplicity and are susceptible to performance and reporting bias in an unblinded trial, these findings should be regarded as hypothesis-generating only. No safety signals were identified.

As a compound traditional Chinese medicine formulation with multi-target characteristics, NXT integrates the TCM therapeutic principles of promoting blood circulation, removing blood stasis, and unblocking collaterals to relieve pain (Zeng et al., 2021; Gao et al., 2018). Modern pharmacological studies have confirmed that its components, including Astragalus membranaceus, Salvia miltiorrhiza, and Ligusticum chuanxiong, possess multiple mechanisms of action such as antiplatelet aggregation, improvement of endothelial function, and inhibition of inflammatory response (Zhu et al., 2024; Wang et al., 2025; Liu et al., 2017; Meng et al., 2024). These mechanisms may collectively contribute to the improvement of clinical symptoms and outcomes in ACS patients.

Among the exploratory secondary analyses, nominally larger reductions in SAS and SDS scores were observed in the NXT arm compared with control. However, self-reported psychological instruments are particularly vulnerable to expectancy and reporting effects in an open-label trial without placebo control; the magnitude of the observed between-group difference cannot be reliably attributed to a pharmacological effect of NXT and may reflect, in whole or in part, non-specific effects of unblinded treatment allocation. From a mechanistic standpoint, TCM theory of “the heart governing the mind” and modern pharmacological data suggest possible neuroendocrine modulation by NXT components (Liao et al., 2024; Tsybko et al., 2024; Neumann and Landgraf, 2012). Previous studies have demonstrated that NXT in combination with conventional therapy significantly improves HAMD scores, which is consistent with our findings (Han et al., 2019; Huang et al., 2022; Lu et al., 2022). These mechanistic hypotheses warrant confirmation in adequately powered, placebo-controlled, double-blind trials before any clinical inference is drawn.

Nominally greater improvements in the NXT arm were observed across SAQ domains, alongside nominally larger changes in LVEF, LVEDD, and LVESD. These are consistent, but not confirmatory, signals; as with the psychological endpoints, all are self-reported or investigator-measured under unblinded conditions and susceptible to ascertainment bias. Preclinical studies have suggested that NXT may promote angiogenesis and collateral formation (Zhang et al., 2024; Zhao et al., 2022; Hong et al., 2025), which could plausibly underlie improvements in ischemic burden and ventricular remodeling; however, the present study was not designed to test these mechanisms, and the observed echocardiographic differences require independent replication with blinded core-lab image analysis.

Nominally larger reductions in TC, TG, and LDL-C were observed in the NXT arm. This descriptive finding is compatible with, but does not establish, an incremental lipid-lowering effect on top of standard therapy. NXT contains red yeast rice, a source of naturally occurring monacolin K (Shi et al., 2024; Rigillo et al., 2025; Cicero et al., 2023); whether this contributed to the observed lipid differences, or whether the differences reflect residual confounding from unmeasured statin dose adjustment and adherence in an open-label trial, cannot be determined from the present data. Herb-drug interactions with antiplatelet or anticoagulant regimens (aspirin, clopidogrel, ticagrelor, DOACs) were not specifically evaluated and cannot be excluded.

A nominally greater reduction in the TCM syndrome quantitative score and a nominally higher responder rate were observed in the NXT arm. Because the TCM syndrome instrument aggregates patient-reported symptoms (chest discomfort, fatigue, palpitations, emotional symptoms) and is scored by non-blinded investigators, it is subject to the same performance and detection biases as the other subjective outcomes reported here. The concordance of this outcome with other patient-reported measures in the same direction does not constitute independent corroboration, because all such measures are affected by the same bias mechanisms in an unblinded trial and would be expected to move together under performance bias alone. TCM syndrome efficacy is reported here as a descriptive complementary outcome and cannot substitute for hard cardiovascular endpoints such as MACE.

### 4.1 Strengths and limitations

This trial has five interrelated limitations that together preclude any efficacy inference.

First, underpowering. The observed control-arm MACE rate (17.6%) was substantially lower than the 32% assumed in the sample-size calculation. We do not present a *post hoc* power calculation, as such calculations are statistically uninformative once a result has been observed (Hoenig and Heisey, 2001). The MACE result is inconclusive and cannot support any conclusion about MACE reduction with NXT.

Second, unblinded adjudication of a subjective endpoint. Urgent revascularization - a component of the composite - depends on treating-clinician judgment and was not adjudicated by an independent blinded events committee, introducing detection bias that cannot be corrected analytically.

Third, absence of placebo control. For a complex multi-component herbal preparation delivered as identifiable capsules, performance bias is unavoidable and affects adherence, symptom and quality-of-life reporting, clinician thresholds for investigation, and event classification. These mechanisms operate in the same direction as any putative pharmacological effect and cannot be separated from it. This limitation applies to every outcome reported here.

Fourth, no multiplicity adjustment. Multiple secondary outcomes across several domains were analyzed without pre-specified correction; all secondary P values are nominal, and - combined with the second and third limitations - the secondary findings are hypothesis-generating only.

Fifth, complete-case analysis of a rare-event outcome. With 21 events among 152 analyzed participants and 18 losses to follow-up (10.6%), no imputation-based analysis could produce inference that is not dominated by modeling assumptions; we therefore report descriptive event counts with a worst-case sensitivity check (Results 3.2).

Additional considerations include single-country recruitment and incomplete concomitant-medication data (including statin dose and adherence), which further limits interpretation of the lipid findings.

## 5 Conclusion

In this exploratory, open-label multicenter randomized trial, adjunctive Naoxintong Capsule did not demonstrate an effect on major adverse cardiovascular events over 12 months; the primary result is inconclusive and does not support any efficacy claim. Nominally larger changes in the NXT arm were observed for several patient-reported and intermediate outcomes; however, in view of the open-label design, absence of placebo control and blinded event adjudication, lack of multiplicity adjustment, and complete-case analysis of a rare-event endpoint, these findings must be interpreted strictly as hypothesis-generating. Adequately powered, placebo-controlled, double-blind randomized trials with an independent blinded clinical events committee, pre-specified multiplicity control, and intention-to-treat analysis are required before any clinical efficacy of NXT in ACS can be established.

## Data Availability

The original contributions presented in the study are included in the article/Supplementary Material, further inquiries can be directed to the corresponding authors.

## Supplementary material

Supplementary Table S1 Lipid profile before and after treatment.

Supplementary Table S2 Echocardiographic indices before and after treatment.

Supplementary Table S3 Safety laboratory indices before and after treatment.

Supplementary Text S1 Questionnaires & Scoring.
