# Supplementary material for: Naoxintong capsule as adjunctive therapy in ACS: a hypothesis-generating open-label randomized trial
Source: Front Pharmacol. 2026 Aug 4;17:1848036. doi: 10.3389/fphar.2026.1848036 (PMC13481356; doi:10.3389/fphar.2026.1848036)
Supplement: Supplementary file 1 [file Table1.docx]

**Supplementary Table S1. Lipid profile before and after treatment [median (P25, P75) unless noted].**

| Outcome | Time point | NXT group(n=78) | Control group(n=74) | *Z/t* | *P* |
| --- | --- | --- | --- | --- | --- |
| TC(mmol/L) | Baseline | 4.92 (4.31, 5.53) | 4.78 (3.97, 5.51) | −0.909 | 0.364 |
|  | Post-treatment | 3.56 (2.96, 4.02)^**^ | 4.15 (3.85, 4.72)^**^ | −4.883 | <0.001 |
|  | Δ (baseline − post) | 1.45 (0.67, 2.10) | 0.61 (−0.15, 1.39) | −3.934 | <0.001 |
| TG(mmol/L) | Baseline | 1.57 (1.18, 2.13) | 1.60 (1.17, 1.90) | −0.636 | 0.525 |
|  | Post-treatment | 1.23 (0.90, 1.41)^**^ | 1.38 (0.94, 1.86)^*^ | −2.479 | 0.013 |
|  | Δ (baseline − post) | 0.39 (−0.20, 0.96) | 0.18 (−0.37, 0.53) | −2.061 | 0.039 |
| HDL-C(mmol/L) | Baseline | 0.98 (0.84, 1.09) | 0.91 (0.79, 1.09) | −0.948 | 0.343 |
|  | Post-treatment | 1.21 (1.03, 1.41)^**^ | 1.08 (0.97, 1.24)^**^ | −2.347 | 0.019 |
|  | Δ (post − baseline) | 0.23 ± 0.32 | 0.18 ± 0.33 | −1.013 | 0.313 |
| LDL-C(mmol/L) | Baseline | 3.35 (2.66, 3.75) | 2.99 (2.37, 3.67) | −1.438 | 0.151 |
|  | Post-treatment | 2.12 (1.76, 2.69)^**^ | 2.42 (2.03, 2.87)^**^ | −2.359 | 0.018 |
|  | Δ (baseline − post) | 0.94 (−0.29, 1.49) | 0.43 (−0.47, 0.98) | −2.711 | 0.007 |

**Between-group treatment effects on Δ (trial − control):**

| Outcome | Effect size | 95% CI (lower, upper) | Test | P |
| --- | --- | --- | --- | --- |
| TC (mmol/L) | +0.740 | (0.370, 1.100) | Mann–Whitney (HL) | <0.001 |
| TG (mmol/L) | +0.260 | (0.020, 0.520) | Mann–Whitney (HL) | 0.039 |
| LDL-C (mmol/L) | +0.470 | (0.130, 0.820) | Mann–Whitney (HL) | 0.007 |
| HDL-C (mmol/L) | +0.053 | (−0.050, 0.157) | t-test (mean diff.) | 0.313 |

***Note:****Data are shown as median (P25, P75) unless otherwise indicated; HDL-C Δ is mean ± SD due to normality.*

*Within-group comparisons vs baseline: ^**^P<0.001, ^*^P<0.05^**^.*

*For TC, TG, LDL-C, Δ is defined as baseline − post-treatment (a positive value indicates a decrease). For HDL-C, Δ is defined as post − baseline (a positive value indicates an increase).*

*Between-group treatment effects are reported as trial − control: Hodges–Lehmann median differences (HL) for non-normal Δ, and mean difference with 95% CI for HDL-C.*
