# Supplementary material for: Naoxintong capsule as adjunctive therapy in ACS: a hypothesis-generating open-label randomized trial
Source: Front Pharmacol. 2026 Aug 4;17:1848036. doi: 10.3389/fphar.2026.1848036 (PMC13481356; doi:10.3389/fphar.2026.1848036)
Supplement: Supplementary file 2 [file Table2.docx]

**Supplementary Table S2. Echocardiographic indices before and after treatment [median (P25, P75), unless noted].**

| Outcome | Time point | NXT group(n=78) | Control group(n=74) | *Z/t* | *P* |
| --- | --- | --- | --- | --- | --- |
| LVEF | Baseline | 59(47,66) | 57.5(49.75,63) | -0.378 | 0.705 |
|  | Post-treatment | 66(63.75,68)^**^ | 62(57,66.25)^**^ | -4.194 | ＜0.001 |
|  | Δ (baseline − post) | 8(1.75,17.25) | 4(2,9) | -2.923 | 0.003 |
| LVEDD | Baseline | (48.59±5.29) | (48.07±5.05) | -0.622 | 0.535 |
|  | Post-treatment | 41(38,43.25)^**^ | 44(41,46.25)^**^ | -4.154 | ＜0.001 |
|  | Δ (baseline − post) | 7(3,12.25) | 4(0,8.25) | -3.312 | ＜0.001 |
| LVESD | Baseline | 32.5(29,42.25) | 32.5(30,37) | -0.618 | 0.536 |
|  | Post-treatment | 27(24.75,30)^**^ | 29(27,31)^**^ | -3.631 | ＜0.001 |
|  | Δ (post − baseline) | 8.13±8.07 | 3.64±6.59 | -3.748 | ＜0.001 |
| FS | Baseline | 32.04(16.21,37.22) | 32.04(23.42,36.65) | -0.472 | 0.637 |
|  | Post-treatment | 36.36(24.16,41.39)^*^ | 35.35(26.70,37.29) | -1.091 | 0.275 |
|  | Δ (baseline − post) | 5.53±14.71 | 1.85±12.28 | -1.671 | 0.097 |

**Between-group treatment effects on Δ (trial − control):**

| Outcome | Effect size | 95% CI (lower, upper) | Test | P |
| --- | --- | --- | --- | --- |
| LVEF | +4.000 | (+1.000, +7.000) | Mann–Whitney (HL) | 0.003 |
| LVEDD | +3.000 | (+1.000, +5.000) | Mann–Whitney (HL) | <0.001 |
| LVESD | +4.493 | (+2.136, +6.850) | t-test (mean diff., Welch) | <0.001 |
| FS | +3.682 | (−0.653, +8.016) | t-test (mean diff., Welch) | 0.095 |

***Note:****Data are shown as median (P25, P75) unless otherwise indicated (mean ± SD for normally distributed Δ).*

*Within-group comparisons vs baseline: ^**^P<0.001, ^*^P<0.05^**^.*

*For LVEF and FS, Δ = post − baseline (higher is better). For LVEDD and LVESD, Δ = baseline − post (higher is better).*

*Between-group treatment effects are presented as trial − control: Hodges–Lehmann median differences (HL) for non-normal Δ, and mean differences with 95% CI for normally distributed Δ.*
