# Supplementary material for: Naoxintong capsule as adjunctive therapy in ACS: a hypothesis-generating open-label randomized trial
Source: Front Pharmacol. 2026 Aug 4;17:1848036. doi: 10.3389/fphar.2026.1848036 (PMC13481356; doi:10.3389/fphar.2026.1848036)
Supplement: Supplementary file 3 [file Table3.docx]

**Supplementary Table S3. Safety laboratory indices before and after treatment [median (P25, P75), unless noted].**

| Outcome | Time point | NXT group(n=78) | Control group(n=74) | *Z/t* | *P* |
| --- | --- | --- | --- | --- | --- |
| WBC (×10^9/L) | Baseline | 8.83 (7.16, 10.20) | 7.90 (7.30, 9.26) | −1.288 | 0.198 |
| WBC (×10^9/L) | Post | 6.95 (6.44, 7.69) | 7.015 (6.13, 7.58) | −0.144 | 0.886 |
| RBC (×10^12/L) | Baseline | 4.73 (4.46, 5.045) | 4.63 (4.40, 5.00) | −1.047 | 0.295 |
| RBC (×10^12/L) | Post | 4.81 ± 0.44 | 4.70 ± 0.42 | t=−1.615 | 0.108 |
| Hb (g/L) | Baseline | 141.83 (135, 148) | 138.00 (130.13, 145) | −1.822 | 0.068 |
| Hb (g/L) | Post | 145.5 (133.53, 153) | 139.0 (128.96, 147.80) | −1.941 | 0.052 |
| PLT (×10^9/L) | Baseline | 245.32 (212.75, 281.5) | 245.0 (218.98, 279) | −0.009 | 0.993 |
| PLT (×10^9/L) | Post | 237.72 ± 53.91 | 246.11 ± 52.78 | t=0.969 | 0.334 |
| ALT (U/L) | Baseline | 31.5 (21.75, 56.64) | 28.0 (21.75, 37.0) | −1.604 | 0.109 |
| ALT (U/L) | Post | 25.29 (19, 34.83) | 23.57 (19, 28) | −1.300 | 0.194 |
| AST (U/L) | Baseline | 27.19 (18.96, 52.0) | 26.82 (18.0, 42.1) | −0.417 | 0.677 |
| AST (U/L) | Post | 23.0 (19.0, 28.87) | 20.89 (16.97, 30.45) | −1.190 | 0.234 |
| Cr (µmol/L) | Baseline | 85.5 (71.77, 95.25) | 90.0 (78.0, 100.25) | −1.661 | 0.097 |
| Cr (µmol/L) | Post | 90.5 (78.0, 100.53) | 92.0 (75.25, 101.75) | −0.004 | 0.997 |

**Between-group treatment effects on Δ (trial − control): Hodges–Lehmann median differences with 95% CIs:**

| Outcome | HL difference | 95% CI (lower, upper) | P |
| --- | --- | --- | --- |
| WBC (×10^9/L) | −0.44 | −1.04 to 0.32 | 0.222 |
| RBC (×10^12/L) | +0.01 | −0.12 to 0.17 | 0.848 |
| Hb (g/L) | +0.03 | −4.00 to 5.00 | 0.891 |
| PLT (×10^9/L) | −15.00 | −33.00 to 6.00 | 0.173 |
| ALT (U/L) | −0.16 | −5.00 to 2.86 | 0.723 |
| AST (U/L) | +1.00 | −4.00 to 4.15 | 0.719 |
| Cr (µmol/L) | +2.00 | −1.00 to 9.99 | 0.248 |

***Note:****Data are shown as median (P25, P75) unless otherwise indicated (mean ± SD is used where appropriate).*

*Δ is defined as post − baseline for all safety indices.*

*No significant between-group differences were detected in Δ for any index (all P>0.17).*
