# Supplementary material for: Naoxintong capsule as adjunctive therapy in ACS: a hypothesis-generating open-label randomized trial
Source: Front Pharmacol. 2026 Aug 4;17:1848036. doi: 10.3389/fphar.2026.1848036 (PMC13481356; doi:10.3389/fphar.2026.1848036)
Supplement: Supplementary file 4 [file Table4.docx]

**Supplementary Text S1 — Questionnaires & Scoring (SAS, SDS, SAQ, TCM Syndrome Score)**

This file provides English versions of the patient-reported instruments used in the study:

• Zung Self-Rating Anxiety Scale (SAS)

• Zung Self-Rating Depression Scale (SDS)

• Seattle Angina Questionnaire (SAQ) — selected items

• Traditional Chinese Medicine (TCM) Syndrome Score Form

Please respond according to your condition during the past week or past 4 weeks, as specified for each instrument.

**1) Zung Self-Rating Anxiety Scale (SAS) — Past Week**

Instructions: Below are 20 statements. Please read each statement carefully and decide how often you felt that way during the past week. Put a checkmark (✓) in one box for each statement.

Response options for each item: □ None or a little of the time	□ Some of the time	□ A good part of the time	□ Most or all of the time

| **Item** | **None or a little of the time** | **Some of the time** | **A good part of the time** | **Most or all of the time** |
| --- | --- | --- | --- | --- |
| 1. I feel more nervous and anxious than usual. | □ | □ | □ | □ |
| 2. I feel afraid for no reason at all. | □ | □ | □ | □ |
| 3. I get upset easily or feel panicky. | □ | □ | □ | □ |
| 4. I feel like I might lose my mind. | □ | □ | □ | □ |
| 5. I feel that everything is all right and nothing bad will happen. | □ | □ | □ | □ |
| 6. My arms and legs shake or tremble. | □ | □ | □ | □ |
| 7. I am bothered by headaches, neck and back pains. | □ | □ | □ | □ |
| 8. I feel weak and get tired easily. | □ | □ | □ | □ |
| 9. I feel calm and can sit still easily. | □ | □ | □ | □ |
| 10. My heart beats fast. | □ | □ | □ | □ |
| 11. I am bothered by spells of dizziness. | □ | □ | □ | □ |
| 12. I have fainting spells or feel like I am going to faint. | □ | □ | □ | □ |
| 13. I can breathe in and out easily. | □ | □ | □ | □ |
| 14. My hands and feet feel numb and tingling. | □ | □ | □ | □ |
| 15. I am bothered by stomachaches or indigestion. | □ | □ | □ | □ |
| 16. I have to urinate often. | □ | □ | □ | □ |
| 17. My hands and feet are usually dry and warm. | □ | □ | □ | □ |
| 18. My face gets hot and flushed. | □ | □ | □ | □ |
| 19. I fall asleep easily and sleep well through the night. | □ | □ | □ | □ |
| 20. I have nightmares. | □ | □ | □ | □ |

**2) Zung Self-Rating Depression Scale (SDS) — Past Week**

Instructions: Below are 20 statements. Please read each statement carefully and decide how often you felt that way during the past week. Put a checkmark (✓) in one box for each statement.

Response options for each item: □ None or a little of the time	□ Some of the time	□ A good part of the time	□ Most or all of the time

| **Item** | **None or a little of the time** | **Some of the time** | **A good part of the time** | **Most or all of the time** |
| --- | --- | --- | --- | --- |
| 1. I feel down-hearted and blue. | □ | □ | □ | □ |
| 2. I feel best in the morning. | □ | □ | □ | □ |
| 3. I have crying spells or feel like crying. | □ | □ | □ | □ |
| 4. I have trouble sleeping at night. | □ | □ | □ | □ |
| 5. I eat as much as I used to. | □ | □ | □ | □ |
| 6. My sexual functioning is normal. | □ | □ | □ | □ |
| 7. I notice that I am losing weight. | □ | □ | □ | □ |
| 8. I am bothered by constipation. | □ | □ | □ | □ |
| 9. My heart beats faster than usual. | □ | □ | □ | □ |
| 10. I get tired for no reason. | □ | □ | □ | □ |
| 11. My mind is as clear as it used to be. | □ | □ | □ | □ |
| 12. I find it easy to do the things I used to do. | □ | □ | □ | □ |
| 13. I am restless and can’t keep still. | □ | □ | □ | □ |
| 14. I feel hopeful about the future. | □ | □ | □ | □ |
| 15. I am more irritable than usual. | □ | □ | □ | □ |
| 16. I find it easy to make decisions. | □ | □ | □ | □ |
| 17. I feel that I am useful and needed. | □ | □ | □ | □ |
| 18. My life is pretty full. | □ | □ | □ | □ |
| 19. I feel that others would be better off if I were dead. | □ | □ | □ | □ |
| 20. I still enjoy the things I used to enjoy. | □ | □ | □ | □ |

**3) Seattle Angina Questionnaire (SAQ) — Selected Items (Past 4 Weeks)**

Please answer the following according to your chest pain, chest tightness, and angina symptoms during the past 4 weeks.

3.1 Limitation in the past 4 weeks due to chest pain, chest tightness, and angina:

| **Activity** | **Severely limited** | **Moderately limited** | **Mildly limited** | **Slightly limited** | **Not limited** | **Limited for other reasons** |
| --- | --- | --- | --- | --- | --- | --- |
| Putting on your clothes | □ | □ | □ | □ | □ | □ |
| Walking indoors | □ | □ | □ | □ | □ | □ |
| Taking a shower | □ | □ | □ | □ | □ | □ |
| Climbing a hill or stairs (three flights without stopping) | □ | □ | □ | □ | □ | □ |
| Outdoor activities or carrying groceries | □ | □ | □ | □ | □ | □ |
| Walking briskly (about 1 km) | □ | □ | □ | □ | □ | □ |
| Jogging (about 1 km) | □ | □ | □ | □ | □ | □ |
| Lifting or moving heavy objects | □ | □ | □ | □ | □ | □ |
| Vigorous exercise (e.g., swimming or playing ball) | □ | □ | □ | □ | □ | □ |

3.2 Compared with 4 weeks ago, the occurrence of chest pain, chest tightness, and angina during your most strenuous activity is:

□ Markedly increased	□ Slightly increased	□ The same	□ Slightly decreased	□ Markedly decreased

3.3 During the past 4 weeks, the average number of episodes of chest pain, chest tightness, and angina:

□ ≥4 times/day	□ 1–3 times/day	□ ≥3 times/week	□ 1–2 times/week	□ <1 time/week	□ No episodes

3.4 During the past 4 weeks, average use of nitro-medications (e.g., nitroglycerin) for chest pain, chest tightness, and angina:

□ ≥4 times/day	□ 1–3 times/day	□ ≥3 times/week	□ 1–2 times/week	□ <1 time/week	□ None

3.5 How bothersome is it for you to adhere to prescribed medications for chest pain, chest tightness, and angina?

□ Severe	□ Moderate	□ Mild	□ Minimal	□ None	□ No medication prescribed by physician

3.6 Satisfaction with the various treatments for chest pain, chest tightness, and angina:

□ Not satisfied	□ Mostly not satisfied	□ Partly satisfied	□ Mostly satisfied	□ Highly satisfied

3.7 Satisfaction with your doctor’s explanation of chest pain, chest tightness, and angina:

□ Not satisfied	□ Mostly not satisfied	□ Partly satisfied	□ Mostly satisfied	□ Highly satisfied

3.8 Overall satisfaction with the current treatment of chest pain, chest tightness, and angina:

□ Not satisfied	□ Mostly not satisfied	□ Partly satisfied	□ Mostly satisfied	□ Highly satisfied

3.9 To what extent have chest pain, chest tightness, and angina affected the enjoyment of life during the past 4 weeks?

□ Not satisfied	□ Mostly not satisfied	□ Partly satisfied	□ Mostly satisfied	□ Highly satisfied

3.10 If chest pain, chest tightness, and angina continue in your future life, how would you feel?

□ Not satisfied	□ Mostly not satisfied	□ Partly satisfied	□ Mostly satisfied	□ Highly satisfied

3.11 How worried are you about heart attack and sudden death?

□ Always worried	□ Often worried	□ Sometimes worried	□ Seldom worried	□ Never worried

**4) Traditional Chinese Medicine (TCM) Syndrome Score Form**

For each symptom below, please select the level that best describes your condition.

| **Symptom** | **None (0)** | **Mild (1)** | **Moderate (2)** | **Severe (3)** |
| --- | --- | --- | --- | --- |
| Chest pain | □ None | □ Mild stabbing pain, relieved spontaneously | □ Marked stabbing pain, requires medication | □ Severe persistent pain, not relieved |
| Chest tightness | □ None | □ Slight chest tightness | □ Obvious, with occasional sighing | □ Asphyxiating chest tightness, frequent sighing |
| Palpitations | □ None | □ Occasional, mild discomfort | □ Intermittent, obvious discomfort | □ Frequent, affects daily life |
| Shortness of breath | □ None | □ After general activity | □ After slight activity | □ Even at rest |
| Fatigue | □ None | □ Tired but can manage work and life | □ Generalized weakness, barely able to work | □ Severe fatigue, unable to sustain daily work |
| Spontaneous sweating | □ None | □ Skin slightly moist at baseline, worse with slight activity | □ Skin moist at baseline, sweats with slight activity | □ Sweats at baseline, profuse with activity |
| Cyanotic lips | □ None | □ Slightly dusky | □ Purple-cyanotic | □ Dark purple-cyanotic |
| Other (specify) | □ □ | □ □ | □ □ | □ □ |
| Other (specify) | □ □ | □ □ | □ □ | □ □ |

Tongue and Pulse Examination

| Tongue body (select all that apply): | □ Normal □ Enlarged □ Small □ Old □ Tender □ Pale □ Purple □ Dusky □ Red □ Stasis spots □ Stasis speckles □ Teeth-marks □ Other: ________ |
| --- | --- |
| Sublingual veins: | □ Tortuous □ Distended □ Purple-red □ Dark purple □ Other: ________ |
| Tongue coating: | □ Normal □ Thick □ Thin □ Scant □ Peeled □ None □ White □ Yellow □ Greasy □ Scant fluids □ Other: ________ |
| Pulse: | □ Normal □ Deep □ Slow □ Rapid □ Thready □ Wiry □ Weak □ Slippery □ Choppy □ Rapid-irregular □ Knotted □ Intermittent □ Other: ________ |
